# Desalination and Detoxification of Textile Wastewater by Novel Photocatalytic Electrolysis Membrane Reactor for Ecosafe Hydroponic Farming

**DOI:** 10.3390/membranes12010010

**Published:** 2021-12-23

**Authors:** Muhammed Iberia Aydin, Damla Ozaktac, Burak Yuzer, Mustafa Doğu, Hatice Inan, Hatice Eser Okten, Serdar Coskun, Huseyin Selcuk

**Affiliations:** 1 Department of Environmental Engineering, Faculty of Engineering, Istanbul University-Cerrahpasa, Avcilar, Istanbul 34320, Turkey; damlaozaktac@gmail.com (D.O.); byuzer@iuc.edu.tr (B.Y.); hselcuk@istanbul.edu.tr (H.S.); 2 Research Department, MIR-ARGE Coop, Esenyurt 34522, Turkey; mustafadogu@mirarge.com; 3 Environmental Engineering Department, Engineering Faculty, Gebze Technical University, Gebze, Kocaeli 41400, Turkey; 4 Environmental Engineering Department, Engineering Faculty, Izmir Institute of Technology, Gulbahce, Urla, Izmir 35430, Turkey; haticeokten@iyte.edu.tr; 5 Gazi State Hospital, Samsun 55200, Turkey; drsrdrcskn55@gmail.com

**Keywords:** photoelectrocatalytic membrane reactor, textile wastewater, wastewater reuse, hydroponic farming, desalination, toxicity

## Abstract

In this study, a novel photoelectrocatalytic membrane (PECM) reactor was tested as an option for the desalination, disinfection, and detoxification of biologically treated textile wastewater (BTTWW), with the aim to reuse it in hydroponic farming. The anionic ion exchange (IEX) process was used before PECM treatment to remove toxic residual dyes. The toxicity evaluation for every effluent was carried out using the *Vibrio fischeri*, Microtox^®^ test protocol. The disinfection effect of the PECM reactor was studied against *E. coli*. After PECM treatment, the 78.7% toxicity level of the BTTWW was reduced to 14.6%. However, photocatalytic desalination during treatment was found to be slow (2.5 mg L^−1^ min^−1^ at 1 V potential). The reactor demonstrated approximately 52% COD and 63% TOC removal efficiency. The effects of wastewater reuse on hydroponic production were comparatively investigated by following the growth of the lettuce plant. A detrimental effect was observed on the lettuce plant by the reuse of BTTWW, while no negative impact was reported using the PECM treated textile wastewater. In addition, all macro/micronutrient elements in the PECM treated textile wastewater were recovered by hydroponic farming, and the PECM treatment may be an eco-safe wastewater reuse method for crop irrigation.

## 1. Introduction

In recent years, drought, population growth, and industrialization have caused problems in providing access to clean water worldwide. Currently, over 3 billion people worldwide are affected by water scarcity, and the amount of fresh water per capita has decreased by a fifth in twenty years. Hence, over four billion people face severe water scarcity worldwide [1,2]. This has become a critical global issue in many countries, including some European ones [3]. Reusing wastewater by implementing cheaper and more effective treatment methods is a more convenient alternative to water scarcity. Thus, there has recently been increasing interest in wastewater (WW) utilization for various purposes. Its utilization in agricultural food and hydrogen production is of great importance as it agrees with the six main pillars of sustainable energy systems: (i) better efficiency; (ii) better cost-effectiveness; (iii) better resources use; (iv) better design and analysis; (v) better energy security; and (vi) better environment [4]. Renewable energy-based integrated systems offer advantages with multiple outputs while reducing overall energy demand, system cost, and emissions, significantly improving overall efficiencies and hence output generation rates [5]. Agriculture is the primary water consumer in the many Member States of the European Union, accounting for around 33% of average water use, with food crop irrigation having the largest share. The reuse of appropriately treated WW has been an essential source for irrigation as it is a potential nutrient source for crops and reduces freshwater demand [6,7].

On the other hand, emerging pollutants and pathogens may be present in the secondary treated WW and eventually pose a risk in the food chain [3,8,9]. However, there is still no scientific consensus regarding the actual level of risk associated with micropollutants and pathogens in wastewaters. Therefore, advanced treatment technologies are to be undertaken for the safe use of reclaimed WW [6].

The textile industry is a critical water consumption sector that produces massive amounts of wastewater, including many toxic compounds [10]. Chemical and aerobic biological treatment methods are two main processes used to treat textile wastewater before discharging its effluent in the receiving environment. However, most dyes used in the textile industry are non-biodegradable. Therefore, textile wastewater may be toxic even after biological treatment. Advanced oxidation processes (AOPs) and membrane processes have proven potential over other biological and adsorption processes to remove toxic compounds from secondary treated textile wastewater [11]. However, selecting advanced treatment methods depends on investment, operational costs, and intended water reuse. The energy and chemical requirements of AOPs are high. Moreover, textile wastewaters are brackish wastewater and a desalination process is required for reuse [12,13]. Membrane processes such as reverse osmosis (RO) and electrodialysis (ED) are the only known practical desalination methods to reuse textile wastewater. However, the investment and operational cost of these processes are high, and the concentrate solution generated during the operation causes another hazardous problem to the environment [14,15]. Consequently, applying membrane processes to reuse textile wastewater is not environmentally friendly.

Solar-based photocatalysis has emerged as a process that can solve the world’s energy and environmental problems with an economical and sustainable approach. Due to the ability of photocatalysis to work with solar energy, it is considered a promising simultaneous eco-safe water treatment and hydrogen production technology in terms of protecting environmental resources. Photocatalytic technology has been developed over the past decades and studied on environmental application areas, such as water treatment [16,17,18,19,20], air purification [21], disinfection [22,23,24,25], hazardous chemical treatment [20,26,27], hydrogen production [28,29,30,31], environmental remediation [32], CO_2_ photoreduction to fuels [33,34,35,36], suitable organic syntheses [37,38,39,40], and renewable energy [41,42,43]. The basic principle of utilizing sunlight energy is the absorption of light and the creation of electron-hole pairs by spatial separation. Developing superior catalysts, reduction of electron-hole pairs during photocatalytic treatment, shifting the absorption range of photocatalyst from UV range to the visible range, developing efficient coating methods, and increasing the surface area of photocatalyst coated electrodes are the main challenges to increase the efficiency of photocatalysis for solar water treatment and hydrogen production. The photoelectrocatalytic (PEC) system represents one of the important steps to decrease hole-electron recombination by using a small external electrical potential to improve the performance of the solar-based photocatalytic systems [44,45]. Many works have demonstrated that the degradation of pollutants and hydrogen production rates are higher in the PEC system compared with the photocatalytic process. Chlorine generation on the photoanode differs between the PEC reactor system and the photocatalytic one [46]. Thus, in recent years, PEC systems have also been studied for the disinfection of pathogen-contaminated waters. It was reported that the application of a relatively low positive bias to a photoanode significantly increased the disinfection efficiency of the photocatalyst in the PEC system. The PEC reactor system has demonstrated high potential in waste-to-energy applications [44,47]. Thus, many studies attempted to increase the surface area of the coated catalyst in a PEC reactor system to increase the performance of the PEC system for water treatment and hydrogen production [29,30,48]. However, while PEC systems are suitable for color and organics removal, ion separation cannot be achieved. Therefore, in this study, PEC and another electrochemical process, ED, are integrated to overcome the disadvantages of both systems. ED is also an electrochemical method to separate ions in different solutions.

On the other hand, ED is not designed to treat organics. Therefore, a system that can simultaneously treat organic matter and ions has been developed by combining the PEC system and the electrodialysis membrane stack [49]. Domestic wastewater reuse for irrigation has well been studied in the literature, even at pilot field scale. Textile wastewater is usually treated as a domestic-industrial mixed stream. However, there is limited work on the reuse potential of a complex industrial mixed stream. To the best of our knowledge, no study has been conducted to investigate the reuse potential of textile wastewater for greenhouse crop irrigation in hydroponic systems. In this study, a novel PEC membrane reactor was used for the desalination and treatment of secondary treated textile wastewater for reuse in hydroponic farming.

## 2. Materials and Methods

### 2.1. Analytical Methods

The analytical methods were based on the Standard Methods (SM) [50]. The following parameters were measured: pH (SM 4500-H^+^B); alkalinity (SM 2320 B); COD (SM 5220 D); BOD_5_ (SM 5210 B); TOC (SM 5310-B); total nitrogen (4500 N B); NH^+^_4_ (SM 4500-NH_3_ C); TDS/conductivity (electrometric method); NO_3_^−^, NO_2_^−^ (ion chromatography-SM 4110); Ca^2+^, Mg^2+^, K^+^, Na^+^, Cl^−^ (ion chromatography-EPA method 300.7); total phosphorus (4500 P); chlorine (SM 4500 Cl.G); boron (SM 4500 B); copper, zinc, iron (atomic absorption (SM 3111-B)); TDS (SM 2540 C); acute toxicity test (*V. fischeri*) (Microtox Bioassay Testing System-ISO 11348-3:2007); total coliform (SM: 9922); color ((Pt-Co), (ASTM 1209), Hach DR5000 UVV (Hach, London, ON, Canada) is spectrophotometer was used for absorbance measurements.

### 2.2. Characterization of the Wastewater

In this study, biologically treated textile wastewater (BTTWW) was taken from the discharge point of a synthetic-cotton textile biological treatment plant, located in Denizli, Turkey. The characteristics of the effluent of textile wastewaters are very well studied and were reported earlier [11]. The characterization of the BTTWW is given in Table 1. The low organic contents in the effluent of the biological treatment plant (BOD_5_/COD: 0.23) suggest a poorly biodegradable stream with a high color level that poses major environmental concerns. The high color intensity can decrease the penetration of light essential for aquatic ecosystems. Overall, the wastewater was characterized as a complex stream containing toxic organic molecules. Total nitrogen, total phosphorus, potassium, magnesium, calcium, molybdenum, and boron in the BTTWW are necessary macro- or microelements in the nutrient solution for crop production under hydroponic conditions. Nitrogen and phosphorous are the essential primary nutrients in the BTTWW.

### 2.3. Design of Novel Photoelectrocatalytic Membrane Reactor and Experimental Setup

**Figure 1:**
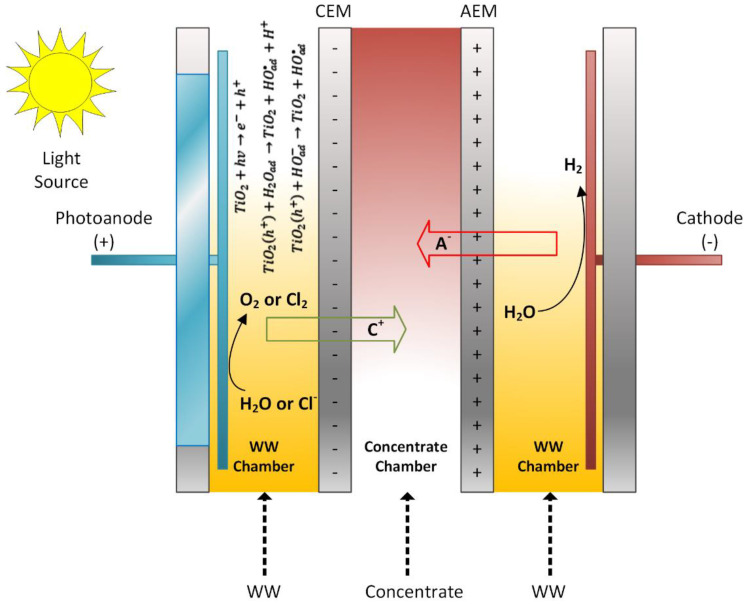
Reactor operation with the PECM reactor.

Two existing technologies, namely electrodialysis (ED) and photocatalysis, have been combined to develop a novel photoelectrocatalytic membrane (PECM) reactor (Figure 1). The designed PECM reactor was composed of three chambers, namely an anode chamber that housed a nanofilm coated photoanode, a cathode chamber, and a middle chamber that produced dilute or concentrate based on the membrane stacking order. Three chambers were separated by ion-exchange membranes, anion exchange membrane (AEM), and cation exchange membrane (CEM). The reactor was made of polypropylene material to support resistance to the deformative effects of heat and chemicals. Characteristics of the used membranes are given in Table 2.

**Figure 2:**
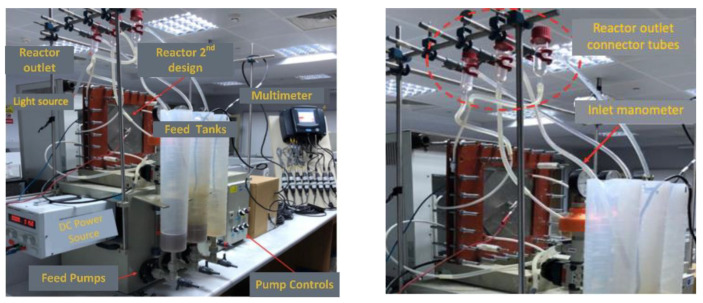
Images of the PECM reactor setup.

The photocatalytic feature was added to the anode compartment and operated as a photoanode using a titanium plate coated with titanium dioxide nanoparticles in the anode compartment (Figure 2). Stainless steel grade 316 L was used as cathode material. The electrodes used in the reactor were 200 × 200 mm. Accordingly, the electrode area was calculated as 400 cm^2^ and the active membrane area was 462 cm^2^. Quartz was used in the anode compartment to pass UV light on the photoanode. UV light was applied to the photoanode during the reactor’s operation using a solar simulator (Atlas Suntest). In this way, TiO_2_ nanoparticles reacted with sunlight to form hydroxyl radicals and enable oxidation in the reactor. The photoanode and cathode were connected via a circuit through a potentiometer. Furthermore, the photoanode chamber was aerated to facilitate photocatalytic reactions and ensure mixing.

The sol-gel method was employed to prepare TiO_2_ nanoparticles for the photoanode. First, 25 mL titanium isopropoxide (TTIP) and 5 mL acetic acid (CH_3_CO_2_H) were added to 500 mL distilled water. Then, 3.5 mL nitric acid (HNO_3_) was added, and the mixture was heated at 80 °C for 30 min. Next, titanium plates of different sizes were dipped into TiO_2_ sol after being cleaned with distilled water and ethyl alcohol. Finally, coated plates were calcinated at 400 °C, and photoactive nanofilm-coated electrodes were obtained.

### 2.4. Testing of the Designed PECM Reactor

Laboratory testing of the designed PECM reactor was carried out with a solution with 2 g L^−1^ NaCl. Laboratory grade NaCl was obtained from Sigma Aldrich (Darmstadt, Germany). All chambers were operated with the prepared solution. The pH, conductivity, and TOC change were monitored under 1 V, 3 V, 5 V, and 10 V applied potentials to test the response of the reactor to change in the potential.

### 2.5. Hydroponic Conditions

**Figure 3:**
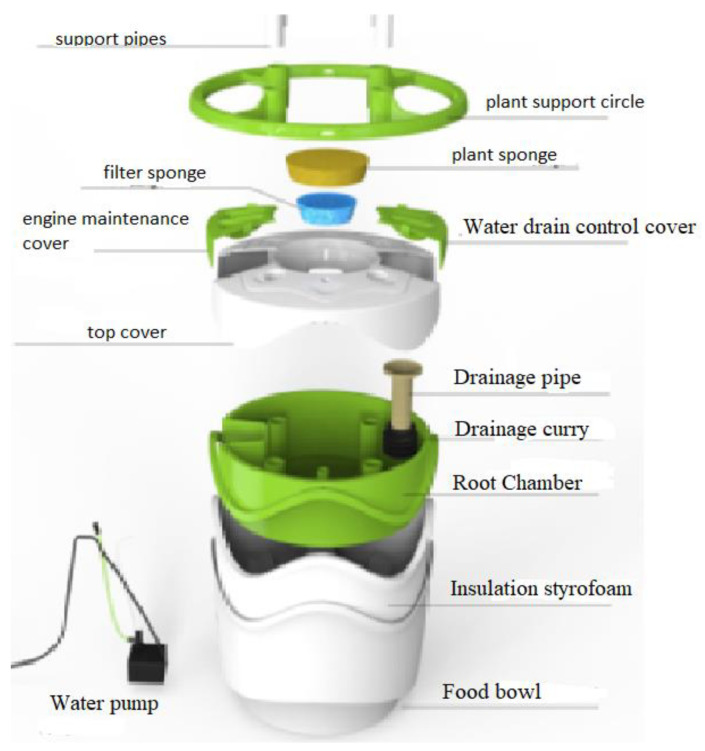
Illustration of the setup of the hydroponic system (Miracle Home Pot).

The hydroponic experiments were carried out at the MIR-ARGE hydroponic laboratory, located in Esenyurt, Turkey of MIR-ARGE company (Turkey). The hydroponic cultivation was conducted once in three replicates. The hydroponic system called Miracle Home pot is where the lettuce was cultivated, as seen in Figure 3. The dissolved oxygen required for plant root systems was provided by aquarium pumping equipment. Lettuce was grown simultaneously using tap water (TW), BTTWW, and diluted PECM treated (pre-treated by anionic exchange method) (IEX+PECM) wastewater under optimum conditions (pH, electrical conductivity (EC), temperature, light, humidity, etc.). The PECM treated wastewater was diluted in order to meet the national reuse standards for irrigation in Turkey. The Miracle Home pot hydroponic system was kept in a well-ventilated condition and at an appropriate temperature (at daytime 23–27 °C and night temperature is below 15 °C). Lettuce could be exposed to sunlight for 8–10 h during the daytime, but lettuce needs 14–16 h of light. Therefore, artificial light was used after daylight. The appropriate macroelements (nitrogen, potassium, magnesium, calcium, phosphate) and microelements (copper, iron, manganese, zinc, boron, molybdenum) were added to the reclaimed water as reported in the literature [8]. The pH and EC values of the nutrient solution to be used for Lettuce are as shown in Table 3. The pH and EC of nutrient solution were measured daily. The pH value was adjusted by adding acid or base to keep it within the range specified in Table 3, and the EC value was adjusted by adding tap water or wastewater. pH and EC were monitored every day, ensuring they were at the desired levels. In addition, the weight and length of the plants were monitored in pots for 80 days to evaluate the effect of wastewater reuse. The results were submitted to an analysis of variance applying the statistical program MSTAT-C, and for the distinction between averages, the least significant difference (LSD) test was used with a 95% confidence range [51].

## 3. Results

### 3.1. Testing of the Novel PEC Membrane Reactor under Different External Potential

The developed PECM reactor was applied for both the treatment and desalination of brackish wastewaters. The PECM system consisting of a membrane stack containing cation (CEM) and anion-exchange membranes (AEM) allows the passage of ions under an external potential. As the PECM process is a combination of photocatalytic and ED processes, the desalination performance of the PECM process depends on the transport of ions by electrostatic and repulsion forces created by the applied potential [44]. Like the ED process, under external potential, transferred negatively and positively charged ions were collected in the middle chamber of the PECM reactor [52]. Thus, the conductivity change in the middle chamber was monitored to evaluate the desalination of the new reactor system. In addition, anode and cathode reactions were separated in a PEC system [48]. Therefore, pH changes in the photoanode and cathode were monitored to evaluate the performance of the reactor under different experimental conditions.

$$TiO_{2}+hv\to h_{VB}^{+}+e_{CB}^{-}$$

on the photoanode

$$H_{2}O+h_{VB}^{+}\to OH+H^{+}$$

and on the cathode

$$2H_{2}O+2e^{-}\to H_{2}+2OH^{-}$$

In the PCEM reactor, chlorine generation and dichlorination occur in the photoanode [48]:

$$2Cl^{-}\to Cl_{2}+2e^{-}$$

$$Cl^{-}+hv\to Cl^{-}+1/2O_{2}$$

**Figure 4:**
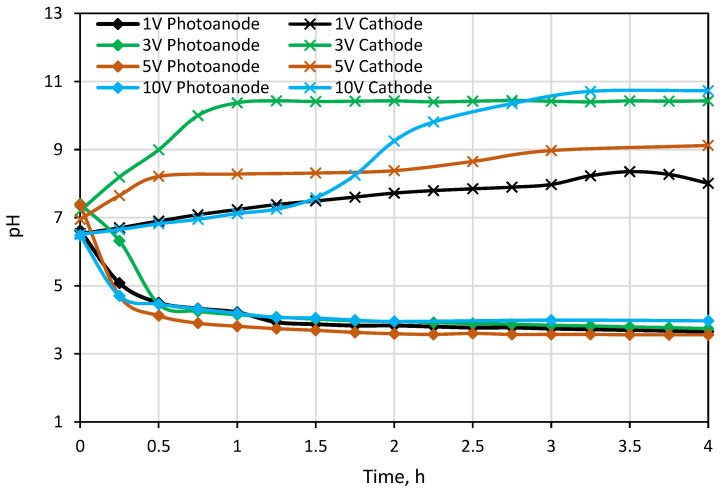
Change in pH values in Photoanode and cathode cell.

**Figure 5:**
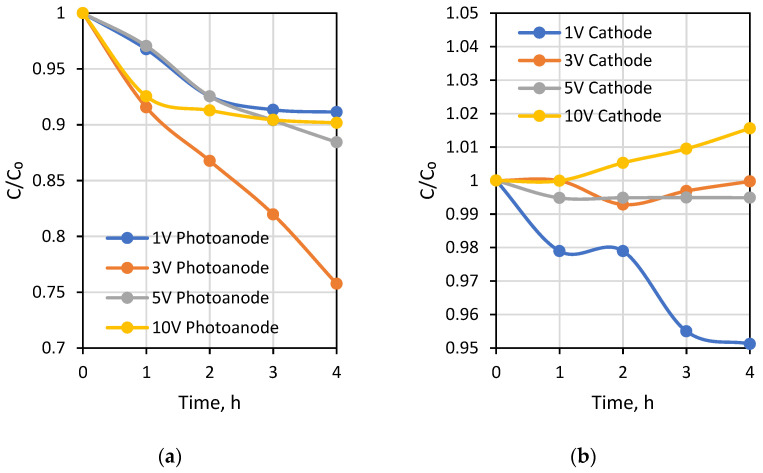
Change of Na^+^ concentration with time in (**a**) photoanode, (**b**) cathode chambers at various applied voltages.

The first testing experiment was carried out under different external potentials to investigate photocatalytic and desalination performances of the PECM reactor system. Due to the separation of hole-electron reactions, pH decreased to around 3.5 in the photoanode compartment and increased to approximately 10 in the cathode compartment during the 1-h PECM treatment period (Figure 4). The pH of the concentrate compartment was stagnant due to the equal ion movement from both sides. The second PECM experiment was performed to investigate the disinfection and desalination performance of the PECM process. The testing solution, including 2000 mg L^−1^ NaCl and 10^9^ CFU E. coli, was treated under various external potentials for 4 h. After 30 min, 2.8 mg L^−1^ chlorine was observed in the reactor. However, chlorine concentration did not change in prolonged periods due to the balance between chlorine generation and dichlorination reactions in the photoanode. The effect of the PECM system on E. coli inactivation has been studied before. In the presence of NaCl, complete deactivation occurs after 45 min with the formation of chlorine [47]. Therefore, in this study, E. coli was measured at 45 min, and inactivation was observed. Rapid and highly efficient disinfection was observed against E. coli. This result was attributed to chlorine generation, UV exposure, and a sharp pH drop in the photoanode. Figure 5 displays the desalination performance of PECM treatment. The number of transferred ions collected in the middle chamber increased slowly and linearly over time and never reached a steady-state condition during the 4 h. Moreover, the turbidity of the treated solution was increased at 5 V and 10 V applied potentials.

Further testing showed an iron interference. This is attributed to the corrosion of stainless steel electrodes [53]. Therefore, tests with real wastewater at 5 V and 10 V applied potentials were not continued. In this work, we used a two-dimensional (2D) electrode. As desalination depends on the current density and surface area of membrane stacks [13], further studies should be done by using three-dimensional (3D) electrodes that have a higher surface area [54].

### 3.2. Pilot Scale Application of PEC Membrane Reactor for Reuse of Secondary Treated Textile Wastewater

**Figure 6:**
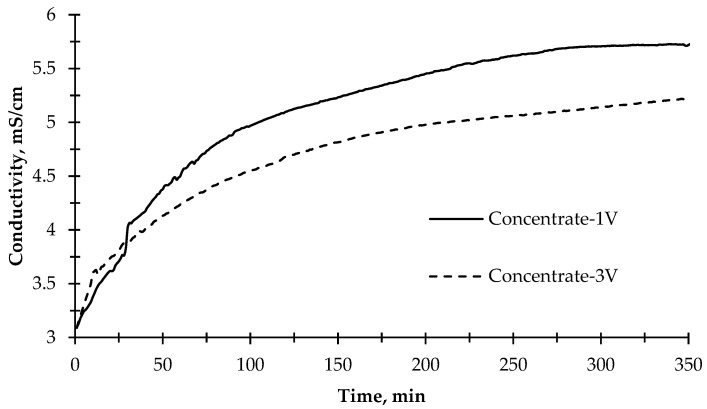
The change of electrical conductivity of the concentrate solution during the desalination experiment with 1 V and 3 V applied potentials and illuminated with the sun simulator.

The change of electrical conductivity values of the concentrate solution, as given in Figure 6, during the desalination experiment showed that the rate of change in conductivity slowed down after approximately 100 min for both applied potential values (1 V and 3 V). The ion transfer rate in the first 120 min was calculated as 8.3 mg L^−1^ min^−1^. However, the ion transfer rate through the membranes for 350 min was calculated as 3.73 mg L^−1^ min^−1^ on average when 1 V potential difference was applied. On the other hand, when 3 V was applied to the reactor, the concentration compartment’s average ion transfer rate was 2.5 mg/L/min. In the first 120 min, the ion transfer rate to the concentrate compartment was calculated to be 5.3 mg L^−1^ min^−1^, which decreased compared with the 1 V run. The reason for this might be that when a high potential difference is applied, the reactor acts like an electrolysis reactor, and more OH^−^ and H^+^ ions are produced, thus passing into wastewater. Instead of other ions, OH^−^ and H^+^ ions passed rapidly to the concentrate compartment and produced water so that the increase of conductivity was limited in the concentrate compartment. At the same time, OH^−^ ions combine with divalent inorganic ions and cause the formation of scaling on the cation exchange membrane, resulting in rapid clogging of the cation exchange membrane [55]. In addition, the photocatalytic reaction efficiency decreased when high voltage was applied. Moreover, the conductivity of the photoanode and cathode cells remained near-constant. Only minor fluctuations were observed. This phenomenon can be explained by the correlation of pH in photoanode and cathode cells. While ion exchange membranes pass the ions that cause conductivity into the concentrate compartment, the remaining ions at the relevant compartments form acid and base, thus decreasing or increasing the pH. Therefore, the conductivity remains constant because of the formation of acid and base.

**Figure 7:**
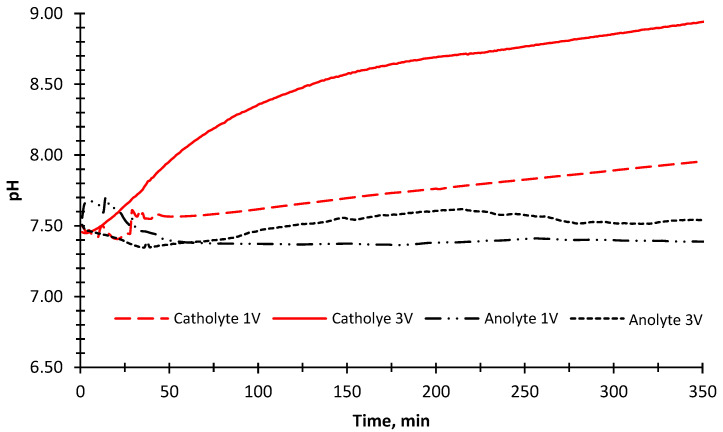
The change of pH values of the anode and cathode solutions (BTTWW) during the desalination experiment with 1 V and 3 V applied potentials and illuminated with the sun simulator.

The pH change was also measured in the anode and cathode compartments during the experiment (Figure 7). An increase in the pH value was observed because OH^−^ ions were being released as a result of converting water into H_2_ gas in the cathode compartment. In parallel with this, the pH value decreased slightly due to the formation of H^+^ ions in the anode compartment in the first 40 min of the experimental run when a 1 V potential was applied. The pH value did not change after 40 min. This could be the fouling of ion-exchange membranes due to the organic matter and inorganic divalent cations [56]. The fouling of the ion exchange membranes prevents the current transfer and decomposition of water into O_2_ gas, thus eliminating the formation of H^+^ ions. The pH results showed an increase in the cathode compartment (Figure 7) when 3 V potential was applied. In the anode compartment, pH values decreased in the first 40 min, and then a steady increase was observed until 215 min. The higher pH value in the cathode compartment compared to the 1 V potential difference was due to the increase in H_2_ gas formation by passing more current through the reactor.

The COD and TOC values of the wastewater treated in the reactor were 510 mg L^−1^ and 165 mg L^−1^, respectively. OH^−^ formed due to the oxidation of organic matter with TiO_2_ nanoparticles in the anode chamber. In the cathode compartment, negatively charged organic dyes were transported to the concentrate compartment over the anion exchange membrane under electric current. During this transport, negatively charged dyestuffs were held on the anion exchange membrane, causing the membrane to clog, changing its physical properties [57]. COD and TOC concentrations of the mixed wastewater (anolyte + catholyte) at the reactor outlet were 246 mg L^−1^ and 60 mg L^−1^ min^−1^, respectively. The reactor demonstrated approximately 52% COD, 63% TOC, and 98% color removal efficiency.

**Figure 8:**
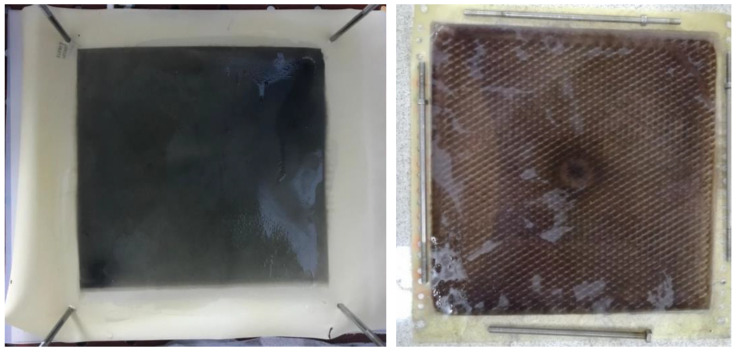
The images of the anion exchange membranes after treatment of textile wastewater without color removal process.

**Figure 9:**
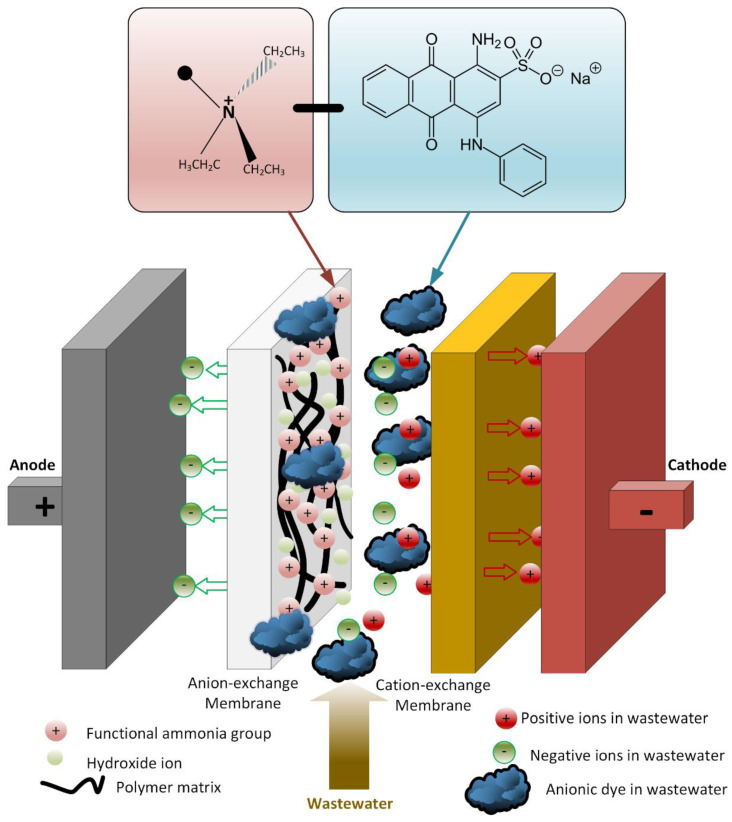
Schematic representation of membrane clogging due to the negatively charged dyestuffs.

There was no oxidation on the cathode electrode in the PECM reactor. In general, the hydroxyl radical, ozone, and oxygen production occurred on the anode, so the removal of color and organic matter was expected in the anode compartment. When the reactor was operated, it was observed that the cation exchange membrane surface facing the anode compartment did not show any color due to dyes, but the anion exchange membrane surface facing the cathode chamber turned into a dark black color (Figure 8). In the textile industry, 75% of the dyes used were negatively charged reactive dyes. Reactive dyes were not oxidized during biological treatment, and their structure remained intact in the sludge and effluent. Therefore, it was concluded that the PECM treatment system needed appropriate pre-treatment processes like other membrane treatment systems. Figure 9 represents the schematic view of the membrane clogging due to the negatively charged dyestuffs. The negatively charged component of the anionic dyes is combined with the positively charged component of the ion-exchange membrane and causes irreversible fouling of the membranes. In this work, we used an anionic ion exchange resin as a pre-treatment of the PECM system to control membrane clogging. Ion-exchange resins can remove ionic dye solutions from wastewater and provide anion to the solution. Then, the anions are removed in the PECM system. On the other hand, another color removal pretreatment method such as ozonation is an alternative for the removal of dye solution. Yuzer and Selcuk [58] have used the ozonation process as a pretreatment for a bipolar membrane electrodialysis system and reported that ozonation might be the solution for ion exchange membrane clogging due to the negatively charged dyestuff. Moreover, the adsorption process might be an alternative color removal method prior to the PECM process since the process does not produce any by-product that can clog the ion-exchange membranes. However, the use of processes which produce by-products or residues that can clog the ion-exchange membranes should be avoided.

The acute toxicity test was conducted for effluents of BTTWW, IEX, and PECM processes. The results of toxicity analyses are given in Table 4. The principle of the test is measuring the light output of the luminescent bacteria after they have been challenged by a sample and comparing it to the light output of a control (reagent blank) that contains no sample. The light output difference (between the sample and the control) is attributed to the effect of the sample on the organisms (Microtox Manual). The BTTWW had toxicity on the *Vibrio fischeri* bacteria at a 60.90% ratio in 5 min, and that ratio was increased to 78.72% after 15 min. Since reactive dyes are toxic [59], the IEX process decreased this toxicity level to 14% and 31% at 5 min and 15 min, respectively. The toxicity level in the cathode compartment did not change during the process. However, the toxicity level at the anode compartment increased to 40% and 57% at 5 min and 15 min, respectively. This increase in toxicity may be attributed to the formation of chlorine generation at the anode compartment (0.28 mg L^−1^) because, after the removal of chlorine, the toxicity level of wastewater was decreased from 78% to 14%.

### 3.3. The Use of PECM Treated Wastewater for Ecosafe-Farming

**Figure 10:**
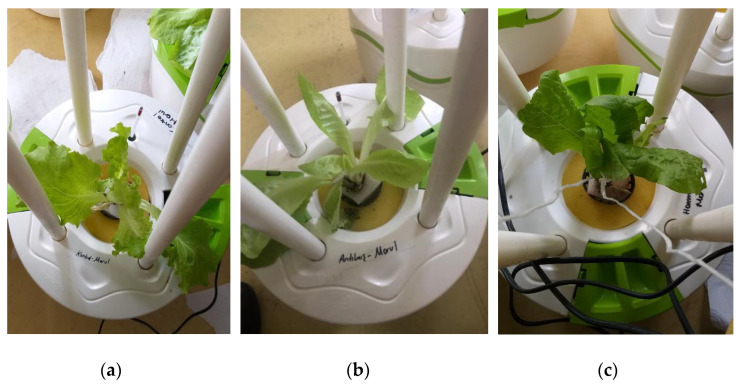
The images of lettuces irrigated with (**a**) tap water, (**b**) PECM treated wastewater, (**c**) BTTWW.

The poor vegetative yield in lettuce (0.26 g average per plant) irrigated with the BTTWW is in agreement with the results found in a similar study [60] in which low yield was reported for lettuce irrigated with diluted treated wastewater. Similarly, there is a significant difference in plant height in the various treatment solutions (Figure 10). In the first and second weeks, the average heights of lettuce plants (8.8 cm) treated by BTTWW were respectively 1.6 cm and 2.2 cm shorter than the other plants, which were irrigated by the effluent of the IEX+PECM treatment processes and tap water. On the 18th day, visible acute toxicity effects were observed on the leaf appearance of all BTTWW irrigated plants (Figure 10c). The BTTWW contains many recalcitrant organics, such as dyes and phenolic compounds. These compounds hinder plants’ access to available nutrients in reused water for good vegetative development and growth [12].

In this work, the stunted growth and detrimental effect on the leaves in BTTWW irrigated plants were attributed to the inhibitory effect of toxic dye compounds in the BTTWW. The height of plants and vegetative yields produced by the effluent of the IEX+PECM treatment processes and tap water were almost the same according to standard deviations. Toxicity data show that colored dyes are the main inhibitory compounds in the BTTWW. Thus, the removal of dyes by the IEX process reduced the toxicity level of treated wastewater from 78.72% to 31.88%. The PECM treatment after the IEX process additionally decreased the toxicity level from 31.88%to 14.56%. The PECM process is an advanced oxidation process and oxidizes or mineralizes many toxic organic compounds [12,61]. The partial or complete breakdown of recalcitrant and complex organic compounds, inactivation of pathogens, and decreasing toxicity and COD levels in treated wastewater are crucial for the reuse of secondary treated textile wastewater in newer farming systems. As the IEX+PECM has great detoxification, treatment, and disinfection potential, it might be a promising polishing treatment for the eco-safe reuse of treated wastewater in hydroponic farming. However, further studies are required to enhance its low desalination performance for the feasible reuse of brackish reclaimed wastewaters.

## 4. Discussion

This study tested a novel PEC membrane reactor in the appropriate environment. Without membrane stack and external potential, the photocatalytic degradation of model organic matter (humic acid) in the reactor was insignificant (around 4.7%), and no pH change and chlorine generation were measured during the 1 h reaction period. However, the membrane stack in the PEC reactor resulted in chlorine generation (0.3 mg L^−1^). In addition, pH dropped from 8 to around 3 in the anode and around 10 in the cathode compartments. Not only chlorine generation, but also rapid pH changes create significant potential for the disinfection of pathogens in the wastewaters. Desalination during the PECM treatment was found to be time-dependent. Thus, efficiency of around 33% was achieved in a 5-h reaction period and this increased over time. The results reveal that the desalination rate in the PECM reactor is low and significant desalination requires a long time and higher electrode surface area. Thus, in subsequent work, a 3D electrode is planned to be utilized to enhance the desalination performance of the PEC membrane reactor.

The IEX+PECM process was applied for detoxification, desalination, and disinfection of real secondary treated textile wastewater. The toxicity level in the BTTWW was reduced by over 75% after the IEX+PECM treatment. Due to the chlorine generation and sharp pH changes in the reactor, the PECM treatment provided great performance for the disinfection of wastewater. The desalination performance of the PECM process is significant, however it reuiqres a long time and better electrodes for a feasible application. Three irrigation solutions of the BTTWW, the effluent of the IEX+PECM treatment processes, and tap water were used to grow the lettuce plans under hydroponic conditions. Detrimental inhibitory effects were observed using BTTWW, while IEX+PECM treated wastewater showed no adverse effect on the growth of lettuce plants under hydroponic conditions. Results indicate that PECM has great potential to reuse industrial wastewater for crop farming. However, these treatment technologies still require a better mass transfer in the reactor, superior photocatalyst and films on the photoanode, efficient electrodes with high surface area, better ion exchange membranes, flexible materials, and reactor designs. Thus, further works should be undertaken to improve the desalination performance of the PECM technique.

Organic matter removal, disinfection, and ion separation can be performed simultaneously with the PECM system. It is also possible to obtain valuable by-products, such as acids and bases. In this case, it is possible not only to treat wastewater, but also to recover valuable products. However, more optimization studies are needed to bring the treated water to a reusable level. These optimization studies are also necessary to improve the existing reactor construction economy. The materials and catalysts used for the electrode and photocatalysis reactions to occur efficiently increase the installation cost of the reactor. PECM is not suitable for commercial usage at its current technological level.

The IEX+PECM processes were applied for detoxification, desalination, and disinfection of real secondary treated textile wastewater. Without the pre-color-removal step, the PECM treatment was at fault due to the fouling effect of negatively charged reactive dyes. The IEX process was used as a pre-treatment to remove color before the PCEM process, and the PECM reactor demonstrated approximately 52% COD and 63% TOC removal efficiency. The toxicity level in the BTTWW was reduced by over 75% after the IEX+PECM treatment. The desalination rate during the PECM treatment with 2.5 mg L^−1^ min^−1^ at 1 V potential was found to be time-dependent. The results reveal the fact that desalination by the PECM reactor requires a long time and/or higher electrode surface area. Thus, in our subsequent work, we will use a 3D electrode to enhance the desalination performance of the PEC membrane reactor.

## 5. Conclusions

In this study, a novel PECM membrane reactor was tested in the relevant environment. The use of a membrane stack in the PEC reactor resulted in chlorine generation (2.8 mg L^−1^). Not only chlorine generation, but also rapid pH changes enabled the reactor to show great potential for the disinfection of pathogens in a short time (in 30 min.). Three different irrigation solutions of the BTTWW, diluted IEX+PECM treated wastewater, and tap water were used to grow the lettuce plants under hydroponic conditions. Detrimental inhibitory effects were observed using BTTWW while diluted IEX+PECM treated wastewater showed no negative effect on the growth of lettuce plants. Furthermore, all macro/micronutrient elements in the PECM treated textile wastewater were recovered by hydroponic farming. Overall, results indicate that PECM has great potential in the reuse of hazardous industrial wastewater for crop farming. However, these treatment technologies still need a better mass transfer in the reactor, superior photocatalyst and films on the photoanode, efficient electrodes with high surface area, better ion exchange membranes, flexible materials, and reactor designs.

## Figures and Tables

**Table 1:** Characterization of biologically treated textile wastewater.

Parameter	Range
pH	5.65–7.75
Conductivity (mS/cm)	6.71–7.65
TDS (g/L)	3.66–4.10
COD (mg/L)	140–600
BOD_5_ (mg/L)	58–180
Color (Pt-Co)	1280–3250
Total nitrogen (mg/L)	9–15
Total Phosphorus (mg/L)	2–4
Potassium (mg/L)	80–105
Magnesium (mg/L)	14–28
Calcium (mg/L)	72–98
Iron (mg/L)	0.35–1.5
Manganese (mg/L)	0.06–0.5
Boron (mg/L)	0.86–1.87
Toxicity (the *Vibrio fischeri*, Microtox^®^ test)	60–100
Copper (mg/L)	<0.01
Zinc (mg/L)	0.31–0.96

**Table 2:** Characteristics of anion and cation exchange membranes.

Membrane Name	Standard Anion Exchange	Standard Cation Exchange
General Use	Standard desalination	Standard desalination
Membrane type	Strong basic	Strong acidic
	Ammonium	Sulphonic acid
Transfer number KCl (0.1/0.5 N) Acid (0.7/3 N)	>0.95	>0.95
Resistance (ohm)	≈1.8	≈2.5
Active Membrane area, cm^2^	400	400
Water content (w.%)	≈14	≈9
Maximum operation temperature, °C	60	50
Thickness, μm	180–220	160–200
Ionic form	Cl^−^	Na^+^

**Table 3:** pH and EC measurement results of treated wastewater.

Parameter	PECM Treated Wastewater (mg/L)
Total Nitrogen	9.79
Total Phosporus	1.5–2
Potasium	80.76
Magnesium	14.25
Calcium	72.63
Iron	0.35
Manganese	0.059
Boron	0.86
Copper	<0.01
Zinc	0.313

**Table 4:** The acute toxicity assessment of the PECM reactor.

		Toxicity	
Sample Location	Description	5 min.	15 min.
BTTWW	Initial	60.90%	78.72%
IEX	Initial	14.55%	31.88%
IEX+PECM	Cathode	14.55%	31.88%
	Anode	40.61%	56.98%
	After chlorine removal (Anode)	11.80%	14.56%

## Data Availability

Data is contained within the article.
